# Variation of Suspended Particles in the Bottom Layer of the East China Sea with Data from Seafloor Observatory

**DOI:** 10.3390/s19235156

**Published:** 2019-11-25

**Authors:** Dinghui Shang, Rufu Qin, Huiping Xu, Changwei Xu, Kelin Sun, Yusheng Zhou

**Affiliations:** 1 Institute of Deep-sea and Engineering, Chinese Academy of Science, Sanya 572000, China; 1410845@tongji.edu.cn (D.S.); xuhp@idsse.ac.cn (H.X.); sunkl@idsse.ac.cn (K.S.); 2 State Key Laboratory of Marine Geology, Tongji University, 1239 Siping Road, Shanghai 200092, China; xcw@tongji.edu.cn (C.X.); 1410844@tongji.edu.cn (Y.Z.)

**Keywords:** East China Sea Seafloor Observatory, LISST 100X, suspended particles, floc size, bottom (boundary) layer

## Abstract

The in situ scattering and transmissometry laser (LISST-100X), equipped with an acoustic wave and current (AWAC) meter and conductivity, temperature, and depth (CTD) instruments over the seabed in the East China Sea, was used to monitor the variation in suspended particles in the bottom sea layer, including particle size distribution (PSD) and volume concentration. The power law approximation was tested to describe the variability in PSD based on the field data. The results show that the power law was robust in processing continuous data, accompanied with the same optimal reference particle size (~63 μm) and little change in the corresponding exponent (~3.4) in both periods. Suspended particles were divided into three types: macroflocs (>133 μm), microflocs (36–133 μm), and single grains (<36 μm). Particle sizes were coarse during the two seasons, with macroflocs representing more than 60% of all the suspended particles, especially in February, when the particle size spectra were usually open-ended. Results from the harmonic analysis method indicate that tidal-induced resuspension and advection are the major reasons for the diurnal dynamics of sediments. Due to the tidal asymmetry in the region, we only found one mode in volume concentration at the moment of maximum velocity. However, the ratios of macroflocs were bimodal, with maximum floods and ebbs in one tidal cycle in February, when the higher mode at the maximum ebbs may be contributed to by the flocculation of finer particles considering the decreasing ratios of finer particles. Due to the enhanced stratification and the clean barrier built up by the Taiwan Warm Current in the southeast corner, the significant daily variation in suspended particles observed in February weakened in September. The influence of waves was uncertain, although the correlation coefficient between significant wave height and volume concentration was about 64% in February.

## 1. Introduction

Suspended particles, including mineral and biogenic particles, are important components of seawater, carrying most of the nutrients and pollutants in the ocean. The concentration, floc size, and constituents significantly influence the optical properties of sea water as well as the marine ecological environment [1]. Their size and density also determine the settling velocity, which in turn plays important roles in the transportation, deposition, and interactions of sediments in the water column [2,3]. Hence, investigating the temporal and spatial variations of suspended particles in mean grain size, volume concentration, and density is necessary to determine their contributions to biogeochemical exchanges in the water column. The particle size distribution (PSD) is a common parameter used in oceanography and sedimentology to characterize marine sediments and the optical scattering of particles [4]. Several mathematical models have been developed to describe PSD, such as the power law, Gaussian distribution, lognormal functions, and the gamma function [4,5]. Among these models, the power law is widely used because it has been found to conform well with many natural processes and laws. It is essential to verify its availability in coastal areas during continuous monitoring.

In estuarine and coastal areas, suspended particles mostly exist in the form of flocs participating in the frequent flocculation and break-up processes that occur due to the dynamic physical environment. Factors, such as particle concentrations, turbulent shear stress, and the amount of sticky organic compounds can affect the flocculation process [6,7]. Since the size and structure of flocs can be easily altered by water turbulence, the traditional sampling analyzing approaches cannot accurately reflect these variations [8]. The development of optical instruments has enabled in situ observations sampling, using transmissometers [9,10,11] and laser backscatters [12,13]. These earlier optical instruments are unable to quantify the effects of the change of particle size on their recorded results, so they usually require continuous calibration. The laser particle size analyzer LISST has been widely used to interpret phenomena occurring in the ocean, having the ability to efficiently determine characteristics of suspended particles in any particular layer [3,4,5,14,15,16,17,18]. Apart from the concentration, particle size was also found to vary with turbulence [16,17,19]. Finer particles (< 63 μm) begin to aggregate into larger particles in relatively slack waters, whereas deposition of coarser flocs may dominate with a high level of turbulence given their loose structure [17]. Accurate mass concentration of suspended particulate matter cannot be obtained through optical properties measured by the LISST because it is usually controlled by particle size, components, and density [9].

Terrigenous input, marine self-generation, and resuspension of unconsolidated sediments on the seabed constitute the main sources of suspended particles in the ocean. In contrast with coastal waters, which are seriously affected by terrigenous input, offshore waters are significantly controlled by sediment resuspension. Zhang et al. showed that particles from resuspension can account for more than 90% of total suspended particles in some areas [20]. The East China Sea (ECS) is a semi-enclosed wide continental shelf sea with a complex hydrological environment, where the concentration of suspended particulate matter is high and distinctly varies with seasonal changes. The dispersal pattern of suspended particles in the ECS has been intensively investigated, as it is crucial for describing the distribution of geo-biochemical materials in the coastal and offshore areas. However, most of the studies are limited to the surface layer [21,22] and only few researches provided insight into the sediment transport in the bottom layer [23]. Many studies were conducted to examine sediment resuspension and transportation in an area from a macro perspective [21,24], with few provisions of detail features. More advanced methods for monitoring and analyzing are needed in current research.

The deployment of seafloor observation networks has provided the basis for long-term in situ monitoring. The first Chinese experimental seafloor observation station, Xiaoqushan, successfully commenced operation in 2009, and the second station located at the east side of Zhoushan, which was the focus in this study, started operation in 2015. Many instruments, such as conductivity, temperature, and depth (CTD); acoustic doppler current profilers (ADCPs); acoustic doppler velocimetry (ADV); acoustic wave and current (AWAC) meter; and seismograph are equipped on the two stations to monitor variations in physical and chemical parameters in the ocean, and provide real-time data for particular scientific research as well as extreme events like earthquakes and tsunamis.

To avoid being influenced by biofouling of bottom instruments, two periods at the beginning of the two operation cycles in 2015 and 2018 were selected to study the variation in the suspended sediments near the sea bottom at the Zhoushan experimental station. We studied the diurnal and seasonal variations in suspended sediments based on the continuous data collected over the sea floor. With the volume concentration at 32 bins obtained by LISST 100X, we analyzed, in detail, how particle sizes vary with tides in the two seasons. Given the irregular semidiurnal tidal feature in the region, harmonic analysis was used to simulate variations in suspended particles. To obtain an overview of the hydrological environment, three expeditions were conducted on the East China Sea in 2018. The measured data were used to explore the seasonal variations in sediment transport in the area. The ability of the power law model to describe the particle size distribution in this region was evaluated with the continuous data as well.

## 2. Material and Methods

### 2.1. Study Area

**Figure 1:**
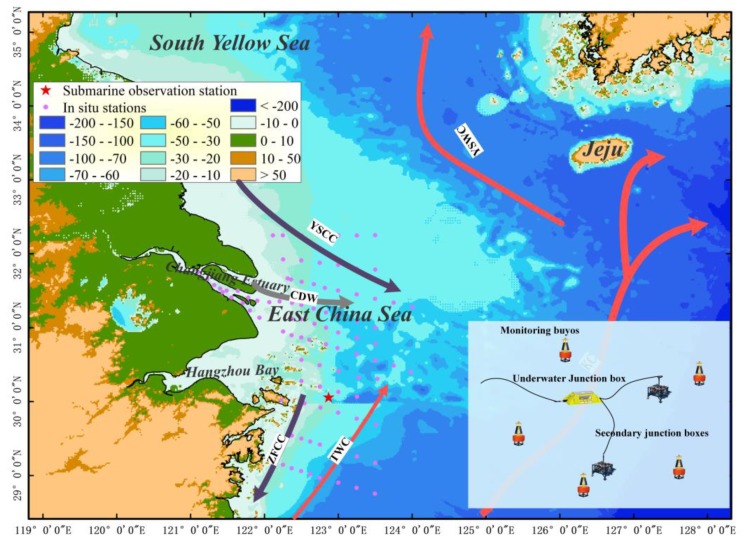
Schematic diagram of circulations (grey and red arrows) in the study area and distributions of observation stations including in situ stations (blue diamonds) and the seafloor observation station (red star); the lower right corner shows the distribution of the seabed-based monitoring platforms.

The East China Sea is a marginal sea in the western Pacific with a wide continental shelf, covering an area of about 1.25 × 10^6^ km^2^. It is seriously influenced by terrestrial sediment input by diluted water from Changjiang River. According to the data measured in the Datong station and published in the East China Sea Marine Environmental Bulletin from 2010 to 2016, the Yangtze River imports about 1.307 × 10^8^ tons of sediment into the East China Sea every year [21]. As shown in Figure 1, subtidal currents in the East China Sea are complex and considerably impact the hydrological environment. The Yellow Sea coastal current (YSCC), Changjiang diluted water (CDW), and Zhejiang–Fujian coastal current (ZFCC) are primary currents in nearshore areas. Since the three currents are influenced by winds, the direction is southward with a strong northeast monsoon in winter that turns eastward or even northward with the moderate southeast monsoon in the summer. In the outer sea, the currents are mainly composed of the Taiwan warm Current (TWC) and Kuroshio Current (KC), flowing northward throughout the year. The intensity of the circulations has significant seasonal variations. The submarine topography is another element controlling the currents’ movements and is colored in Figure 1 using different colors according to the bathymetry data collected from the National Geophysical Data Center (NGDC) with a resolution of 1 arc-minute for both latitude and longitude.

The experimental seafloor observation station is located near the middle of the continental shelf (122°52′12″ E, 30°3′36″ N), with an average water depth of about 41 m. Based on the sedimentary type survey conducted earlier in this area [24,25], the site is adjacent to the muddy area in the Yangtze River estuary and muddy area along the coast of Fujian and Zhejiang provinces in the north and south, respectively. Horizontally, it is dominated by silt and close to the fine sand to the east. Therefore, the location of the sea floor station is favorable for analyzing dynamics of sediment transport near the bottom waters. To avoid the effects of biofouling, we selected a few days early in the two deployments. The first period was between 15 and 20 September 2015, and the second was from 4 to 9 February 2018.

### 2.2. Available Data from Seafloor Instruments

A type C LISST-100X was programmed to monitor suspended particles near the sea bottom, with a measurement range of 2.5 to 500 μm. When the scattered light is measured by the concentric ring detectors in the near forward angles, we can determine the volume concentration of each fraction V (D_i_), where the value of i varies from 1 to 32, with the inversion model derived from the laser diffraction principle. D_i_ indicates the mean grain size of the two measured boundaries of each fraction. In general, it is computed with the assumption that the volume is equivalent to a normal sphere with the given diameter. Then, the V (D_i_) can be calculated according to the script file in MATLAB provided by the Sequoia website [26]. Hence, the number of particles in each size class can be calculated as:

(1) $$N\left(D_{i}\right)\;=\;V\left(D_{i}\right)/\left(\frac{{\pi D}_{i}^{3}}{6}\right).$$

The instrument should be calibrated in clean water before every use to avoid the influence of background on the intensity of received light.

Divided by the total volume concentration of the suspended particles, the relative volume concentration was computed for every size bin for convenience in later analysis. To investigate how the particles with various sizes varied with currents in the water, the 32 bins measured by the LISST instrument were reclassified into three classes: single grains: 2.5–36 μm (bins 1–16), microflocs: 36–133 μm (bins 17–24) and macroflocs: >133 μm (bins 25–32), after several experiments. The result of the classification is consistent with the method proposed by Mikkelsen et al. [27]. Time series of the particle diameters of D25, D50, and D75 were also calculated based on the measured particle size distribution, where D25 is the particle size at which the cumulative concentration reaches 25% of the total. The D50 (also can be called median particle size) and D75 are computed similarly.

The AWAC meter is a current and wave measuring instrument designed on the basis of the acoustic Doppler Effect to provide approximately continuous profiles of current velocity and wave information, such as significant height, direction, and period. It is widely used in marine research, especially in field studies [28,29]. In this study, the AWAC meter was set to burst mode, with a mean value of the measurements transmitted to the data center every 20 min. Given the different settings for the instrument, there were 35 and 20 layers in the vertical direction. With the same vertical space between layers during the two deployments, the corresponding depth of the maximum layer in February 2018 was the same as the 20th layer in 2015. Two Seabird CTD instruments were deployed to measure water temperature, salinity, and depth during long-term observation sessions.

### 2.3. Power Law Approximation of PSD

The PSD is defined as the average number of particles for each fraction with width of Δ*D*_i_ per unit volume ($N^{\prime }\left(D_{i}\right)$), which is expressed as

(2) $$N\left(D_{i}\right)\;=\;N^{\prime }\left(D_{i}\right)\Delta D_{i}.$$

The LISST-100X over the seafloor was sampling 8 times per minute. To eliminate accidental error, as well as reduce the computational cost, the raw data were grouped every 5 min with 40 samples used for median filtering. As proved in earlier studies, the variability in volumetric distribution of particles closely follows a power law expressed as

(3) N′(D)=N′(D0)(DD0)−ζ,

where $D_{0}$ represents the reference particle size, $N^{\prime }\left(D_{0}\right)$ is the corresponding number concentration at $D_{0}$, and ζ is a dimensionless parameter, indicating the slope of the power law model. Hence, the model can be seen as a linear relationship between the ratio of the concentration and the ratio of particle size after logarithmic transformation. Then, we can fit the data using the least squares method to obtain the slope ζ. Some researchers have reported that the slope of the power law can act as a reference criterion to judge whether larger or smaller particles account for the greater proportion of the total particles to some degree: the larger the value of ζ, the more the smaller particles [15,30].

### 2.4. Harmonic Analysis of Tide

The tidal movement of ocean water is caused by the gravitational attraction of the moon and sun acting upon the earth, which can be resolved into a series of simple harmonic motions [31]. Hence, the water depth and current speed can be decomposed according to:

(4) $$Y(t)=\overset{N}{\underset{i=1}{\sum }}{(\alpha }_{i}{\cos w}_{i}{t\;+\;\beta }_{i}{\sin w}_{i}t)$$

where *Y*(t) is the time series of any variable changing over time t; *α_i_* and *β_i_* refer to the amplitudes, which can be calculated by the least squares method; coefficient $w_{i}$ represents the frequency. The process is called tidal harmonic analysis. In this paper, the UTide MATLAB functions provided by Codiga [32] were used to analyze the variation characteristics of water depth, current velocities, and volume concentration of suspended particles over time. All the components were pre-defined by earlier researchers, which can be found on the Wikipedia website [33].

A total of 11 common components (P1, K1, O1, Q1, K2, M2, S2, N2, M4, MS4, and M6) were included in the model. Then, the anomalies and residual current were obtained by subtracting the modeled results from the observed data. The results obtained from sea level and current velocities prove that the M2 tidal component dominates in the station and almost has no deflection within 10 m from the observatory. The rotation rate K, defined as the ratio of the minor axis to the major axis for every tidal current ellipse, is used to evaluate the rotation degree of the tidal current. When its absolute value is larger than 0.25, the tidal current is a rotary current; otherwise, it is a rectilinear current. Comparing with the results in Table 1, the four main components in the layers near the bed are generally rectilinear currents. The percent of tidal variance (PTV) captured by the harmonic analysis was introduced to evaluate its availability. The harmonic results are accurate with a PTV value greater than 90%.

## 3. Results

### 3.1. Particle Size Spectra

**Figure 2:**
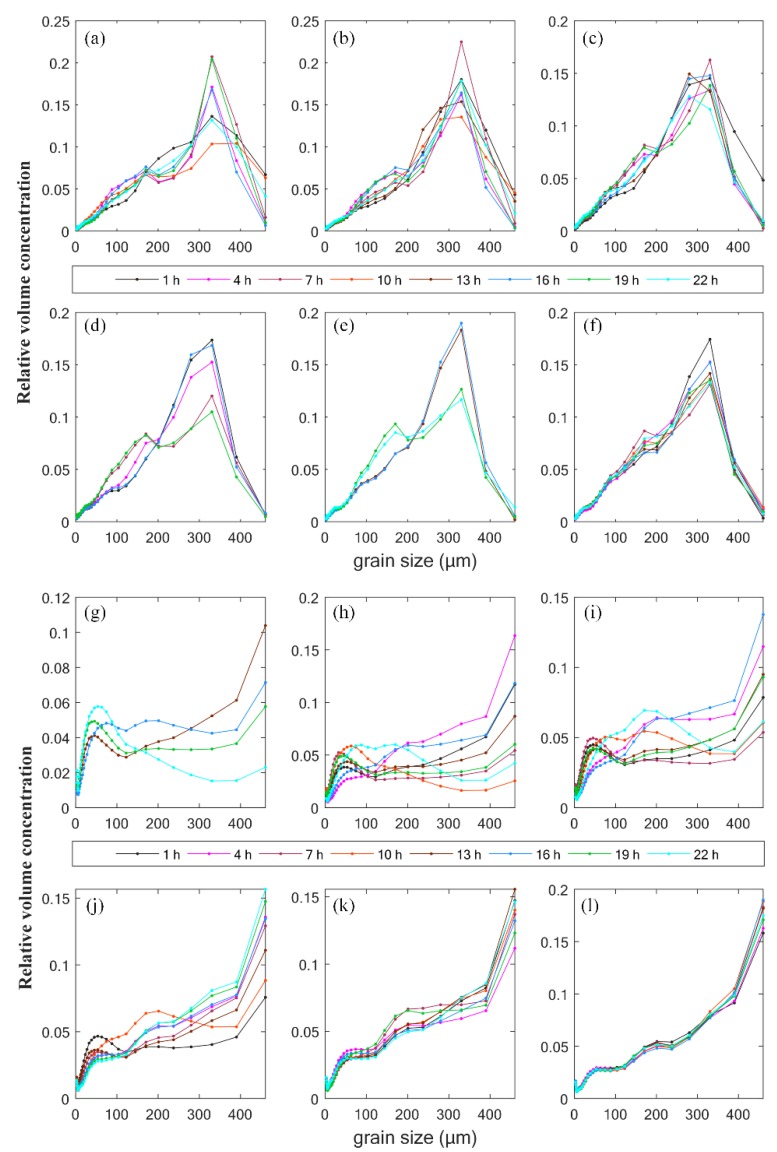
Particle size spectra measured at the submarine observation during (**a**–**f**) September 2015 and (**g**–**l**) February 2018. The relative volume concentration shown on the y-axis has a unit of 1.

The hour averaged relative particle size spectra near the seabed changed obviously over time (Figure 2). To avoid being covered by too many curves in every subplot, only one spectrum was plotted every three hours. Spectra in the two periods showed different patterns. In September 2015, the particle size spectra usually showed a mode around 330 μm (Figure 2a–f). The amplitude of the mode is higher in peak flows than in slack water by more than 20% during maximum flood and ebb. The amplitude was also found to decrease with the tidal range. When the tide turned to flood slack, the amplitude of the mode decreased to the lowest, and particles with size around 280 got similar levels (presented as the curve at the 10th hour). Generally, a submaximal point around 170 μm was observed on the spectra during the deployment, after which spectra began to vary with different tendencies over time. Spectra tended to decrease first and then increase around 200 μm when the velocity was low (Figure 2a,d,e); otherwise, the spectra increased even faster to the mode. The differences among the curves measured every day decreased with the transition from spring to neap tides.

In February 2018, particle size spectra were always open-ended, with the maximum ratio located at the coarsest side. An individual local mode generated around 45 μm in the spectra. Its amplitude showed a tendency to vary with current velocity, reaching the maximum in slack water and the minimum in peak flows. Another point around 133 μm on the spectra was noted during the period. During the spring tide, spectra varied significantly between 133 and 500 μm, with a second mode (~200 μm) appearing when the current velocity was relatively low. The volume ratios of suspended particles varied more significantly during the spring tide than the neap tide in both periods. Diurnal variation amplitude was low in later days (Figure 2f,k,l) with smaller tidal ranges.

Tails shown in the particle size spectra were continually observed during the observation, which was also commonly observed in previous studies [34,35,36]. The tails near the finest size are considered to be caused by the high scattering resulting from the particles smaller than the lower LISST size range [36]. However, the open-ended spectra with the tails around 500 μm indicate the presence of particles coarser than the size range in this period. Limited by the grain size that the LISST instrument can measure, the spectra may not exactly reveal all the suspended particles near the bottom of the water column. Results of earlier attempts showed that parameters calculated from spectra with tails can only be used in similar circumstances to obtain some qualitative results or conclusions about the variations, rather than accurate values [35].

### 3.2. Power Law Approximation

According to Equation (3), slope ζ is not only closely related to the reference particle size *D*_0_ but also to the range of particle size used in the calculation. Several studies found that LISST is sensitive to particles that are finer than the measurement range, which leads to more laser energy being diffracted onto the outer rings [5,19]. In [37], Andrews et al. proposed that the inherent assumption for calculating the refraction index for small particles also impacts the rising tail. Hence, the exponent ζ was computed with the size range between 5.72 and 218.49 μm, corresponding to the ring number 6 to 27 of the instrument, to minimize the inaccuracies caused by the tail and the small number (<50) of coarser particles. The fitting results showed that the power law model is appropriate and robust in the bottom layer of the site, where the relative coefficients for all samples remained greater than 0.95 (*p* < 0.001) in both months. To determine the optimal reference particle size, we introduced the mean absolute relative error (MARE) between the measured and estimated number concentration of suspended particles in logarithmic scale, as shown in Equation (5):

(5) $$\mathrm{MARE}\;=\;\frac{1}{n}\overset{n}{\underset{i=1}{\sum }}\left|\frac{\log_{10}{N(D)}_{es}-\log_{10}{N(D)}_{re}}{\log_{10}{N(D)}_{re}}\right|$$

where $N{\left(D\right)}_{es}$ and $N{\left(D\right)}_{re}$ represent the estimated and observed number concentration of particles, respectively. Given the rings participating in the computation, the value of n in Equation (5) is 22.

**Figure 3:**
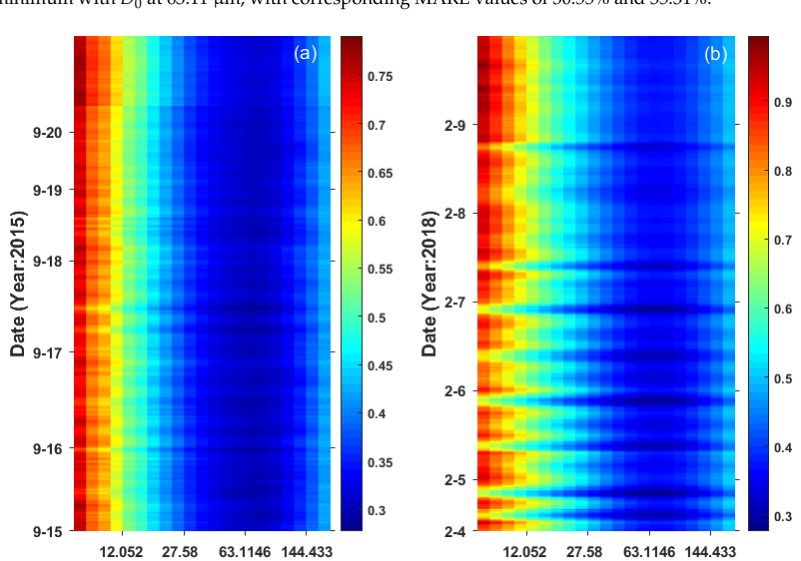
The estimation error of the particle number with a different reference particle size (the vertical coordinates indicate observation time in format of ‘month-day’.

Figure 3 shows the estimation errors of the power law approximation at the site, in which rows represent observation time and columns refer to the selected reference particle size *D*_0_. The invalid diurnal data were eliminated in the figure. The results indicate that the error distribution varied similarly with particle size in the two periods. A too small reference particle size (smaller than 14.22 μm) caused a large estimation deviation, and the MARE at most of the observation times exceeded 50%. In February, the estimation error for small particle sizes varied markedly for the higher ratio of finer particles. The estimation errors of the power law approximations in the two periods both achieved the minimum with *D*_0_ at 63.11 μm, with corresponding MARE values of 30.55% and 35.31%.

### 3.3. Variations in Field Measurements

**Figure 4:**
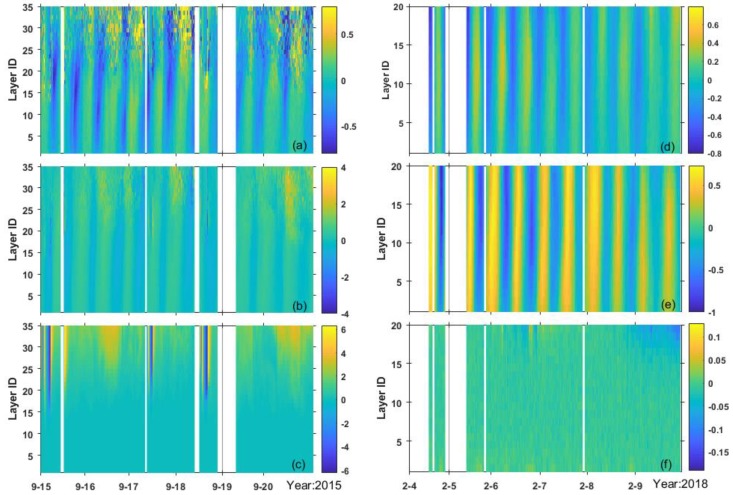
Current velocities at each layer with eastward (**a**,**d**), northward (**b**,**e**), and upward (**c**,**f**) components respectively: (**a**–**c**) velocities in 2015 and (**d**–**f**) velocities in 2018.

The station is located in a high-energy tidal region, where the maximum horizontal current velocity measured at the surface exceeds 2 m/s. As mentioned in Section 2.3, the current velocity obtained in September 2015 covered a higher vertical distance, which nearly reached the surface with 35 layers, but only reached around the middle in February 2018 with 20 layers. The upward velocity seemed to decrease with increasing water depth, getting the maximum velocity of 6 m/s during spring tides near the surface in September (Figure 4c). However, this value was lower than 0.04 m/s near the sea bottom in both sessions, showing little relationship to variations in suspended particles. The upward transport was ignored in later analysis. We found no evident difference between the average velocities measured in the bottom layer during the two periods, and the average value was around 0.32 m/s.

**Figure 5:**
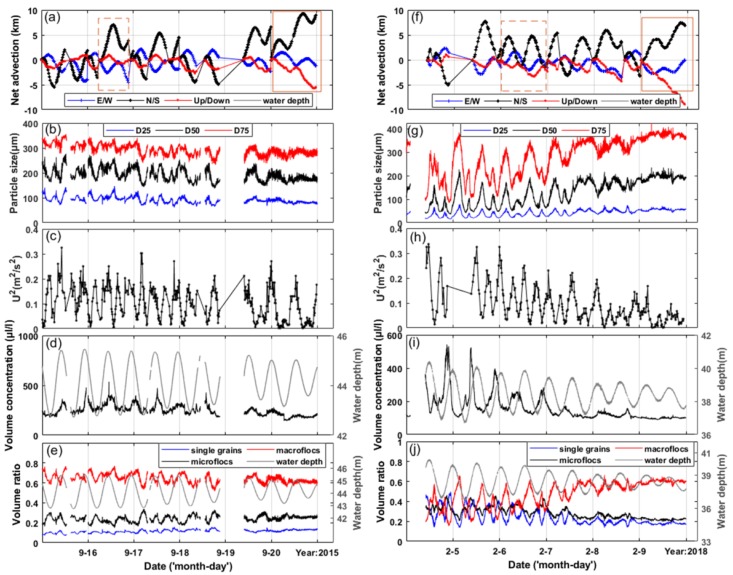
Temporal variation in the field parameters in the two periods, including (**a**,**f**) net advection in three directions, (**b**,**g**) floc size (expressed by D25, D50, and D75), (**c**,**h**) U^2^, (**d**,**i**) water depth, total volume concentration, (**e**,**j**) and volume ratio of single grains, microflocs, and macroflocs.

Theoretically, the turbulent energy production should be calculated from the velocity measurements to quantify the influence of turbulence on floc disaggregation and aggregation [38,39]. However, the sampling interval (~20 min) was too long to obtain an appropriate result. Hence, we used the sum of square velocities in the east and north (U^2^) as the proxy of the current stress [38] to investigate how the particle size spectra vary with tidal processes. The wave-induced near-bed stress was not considered in this strategy, but the effect of the waves is expressed by the relationship between volume concentration or volume ratio of sediments and significant wave heights in later sections. The advective distance in the three directions during the deployment was calculated by the acceleration of the velocity vector measured in each period multiplied by the corresponding time interval vector (Figure 5a,f). Note that when the time interval between two observations of the AWAC meter was longer than one hour, the advection restarted from zero. The vertical advection distances (red lines) were multiplied by 10 before plotting. The advection in the three directions seemed to complete a circle in about 12 h. The advection tended to flow northwest in September and northeast in February, suggesting the different directions of sediment transport to some degree.

To obtain better insight into dynamics of particle size distribution during the two periods, the average value for these parameters was calculated every five minutes. Time series of current velocities, median diameter, and volume concentration of suspended particles are presented in Figure 5. Obvious differences between the two periods were found according to the average values listed in Table 2. In September, suspended particles had a higher absolute volume concentration and a coarser diameter. In February, suspended particles show more dramatic changes, expressed by higher standard deviations. The U^2^ showed a tendency to decrease with the decreasing tidal range in February, which did not occur in September. Since the tidal range varied little during the selected days in September 2015, in contrast with February 2018, the influence of the observation time cannot be ruled out. The floc size, represented by D25, D50, and D75, showed a tendency to decrease as U^2^ increased initially, then increase as U^2^ decreased, and reached the minima during peak flows. Due to the typical semi-diurnal tides observed at the site, the floc sizes were characterized by M4 frequency with four maxima and minima in one day. In addition, the size increased toward neap tides in February, contrary to the trend in September. The findings indicate the complexity of the relationships between the measured environment and suspended particles.

Variations in volume concentrations in each bin were so irregular that detailed comparison tended to be ambiguous. The classification proposed here was tested for generating change rules about particle size distribution. The volume ratio of flocs with diameters coarser than 133 μm generally varied in the opposite direction of flocs smaller than 36 μm in both periods, showing the maximum in peak flows and minima in slack waters. We found that the ratio of macroflocs increased during ebb tides when the absolute volume concentration was significant lower. The volume ratio of the three classes in September was almost constant, slightly fluctuating with time (Figure 5e). However, diurnal variations were evident during spring tides in February (Figure 5j), showing a positive correlation with U^2^ (Figure 5g). Ratios of macroflocs were bimodal with peak flows. When the tides turned to neaps in the latter days of the period, the ratios of the three classes did not vary much and the macroflocs reached the maximum value around 60%, contributing to coarser floc sizes than at other times.

## 4. Discussion

### 4.1. The Adaptability of the Power Law Approximation

The exponent ζ of PSD was relatively stable in the two periods. It varied between 3.02 and 3.32 with a mean value of about 3.18 in September 2015. In February 2018, the exponent ζ had a wider range of 3.11 to 3.90 and a higher mean value (about 3.44), accompanied by a standard deviation of 0.13. The value of ζ is consistent with previous reports stating that the slope ζ in sea waters is usually between three and five [30]. Compared with the water depth monitored by the Seabird CTD, we found that the corresponding ζ during the spring tide was larger than that during neap tide, confirming the conclusions of Bader [4] and Welzzel [15]: the larger the slope, the larger the proportion of small particles in suspended sediments. The higher mean value of ζ found in February also indicates that finer particles accounted for more of the suspended particles, verifying the effectiveness of the law to describe PSD in field work.

### 4.2. Diurnal Dynamics of Suspended Sediments

**Figure 6:**
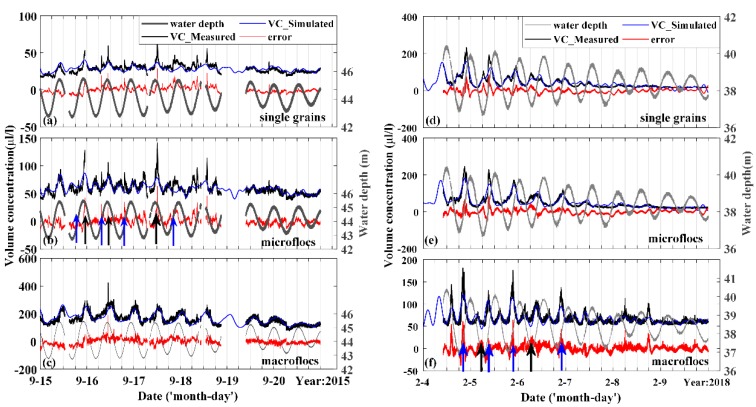
Time series of simulated errors of volume concentration for the three classes in two periods (**a**–**f**). Blue arrows (in spring tides) and black arrows (in slack water) locate at the moments with relatively big estimate errors.

In the ECS, tidal currents play an important role in the variation of suspended sediments distribution with the maximum tidal range reaching about 4.5 m [40]. On the inner shelf near the mouth of the estuary, current-induced bottom shear stresses are usually so strong that they could stir the fine-grained sediments accumulated on the seabed and then transport sediments to the upper waters by vertical mixing [21,24,41]. Located near the central area of the shelf with a lower current speed than the inner shelf, tidal influences on sediments at the seafloor observatory may be restricted to layers near the bottom, according to earlier studies based on satellite observations over the region [21,24]. Since no extreme currents occurred during the two periods according to the measured data (Figure 4), we conducted harmonic analysis with the assumption that variations in the absolute volume concentrations of the suspended particles resulted from tidally-induced resuspension and advection (Figure 6). We introduced the same 11 components into the model and ranked by percent energies (PE). The top four components are listed in Table 3.

The two diurnal tides dominant in September were simulated with similar amplitudes and phases, which could be further simplified into a semi-diurnal tide with double amplitudes within a certain tolerance on the basis of trigonometric functions. Thus, the maximum generally occurred every 12 h during this period, controlled by the highest velocities during maximum ebbs. Similarly, harmonic analysis also explained most of the variation tendency of suspended particles in February. At high tide, the bottom shear stress was enhanced, and a large amount of surface sediment would have been involved in the water column. The advection movement also carried nearby suspended particles through the observation site at the same time.

Figure 5 shows that changes in volume ratios usually occurred with larger flow rates for naturally enhanced resuspension and deposition. Note that not all the peak current velocities cause an increase in volume concentration of suspended sediments, such as the maximum floods in September 2015 and maximum ebbs in February 2018. The tidal asymmetry may be the major reason for this. As it was recorded by the instrument, the velocity in the ebb tides was higher than in the flood tides in September 2015, which was the opposite in February 2018. Due to the higher settling velocity of flocs with high turbulence [42,43], the volume concentration tended to decrease quickly after the maximum ebbs in September, when the resuspension was weak for low current velocities.

**Figure 7:**
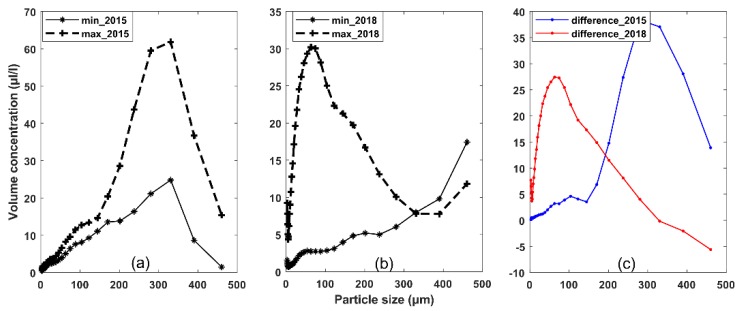
(**a**,**b**) Volume concentration at each bin varied with current velocities during two periods. Solid lines denote spectra measured in peak flows during spring tides and dashed lines denote spectra measured in slack waters during neap tides; (**c**) the difference in the spectra in (**a**) and (**b**).

The typical particle size distribution obtained during peak flows during spring tides and slack waters during neap tides are presented in Figure 7a,b. If the assumption of variation in particle size spectra being only a result of resuspension is reliable, we may infer the size distribution of the surface sediment over the seabed by subtracting the spectra (Figure 7c). Then, the surface sediments in September seemed to be coarser than that in February. However, variations in suspended sediments during peak flows would also affected by: (1) Advection of sediments changing both volume concentration and particle size, depending on characteristics of suspended particles in adjacent areas and (2) disaggregation of flocs lowering particle size with no effect on volume concentration. Hence, surface sediment samples at different seasons will be needed in future analyses to verify our conclusion. The volume concentration of single grains and microflocs did not increase with macroflocs at ebb tides, likely party due to flocculation, which, in turn, explains the second mode for macroflocs.

Since finer particles (single grains and microflocs) are always sensitive to environmental changes, the simulated volume concentration of finer particles often deviated from the true values significantly (moments indicated by arrows in Figure 6), especially in September (PTV < 50%). The underestimation of finer particles (arrows in arrows in Figure 6a,b) in rapid currents is probably promoted by break-up of flocs. Because of the settling out of coarser particles, the volume concentration of sediments in successive slack waters was lower, as well as the median particle size.

**Figure 8:**
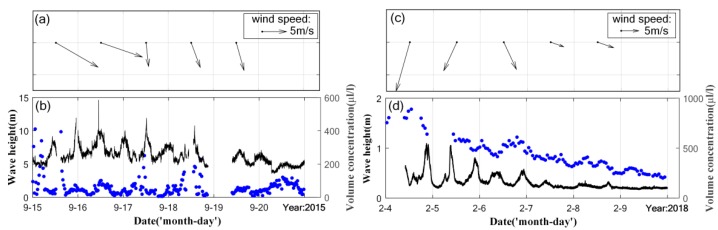
Wind speeds (**a**,**c**) and significant wave heights (**b**,**d**) measured during the two periods.

Influences from wind and waves were also considered in the diurnal dynamics. Wind data were measured using the WindSat Polarimetric Radiometer from space and processed remote sensing systems (RSS), which is sponsored by the NASA (National Aeronautics and Space Administration) Physical Oceanography Program. Two sets of maps contributed ascending and descending passes every day, usually at local time 06:00 and 18:00. Wind and sediment concentration showed very weak correlations (Figure 8), in accordance with the result reported in Bian et al. [44]. Significant wave heights monitored in February showed positive correlations with volume concentration (*R* = 64%), which were unclear in September. The higher measured volume concentration of macroflocs than simulated could be due to waves (blue arrows in Figure 6f). More data are needed to distinguish the effect of waves from tidal currents in future analyses.

### 4.3. Changes in Suspended Sediments between the Flood and Dry Seasons

**Figure 9:**
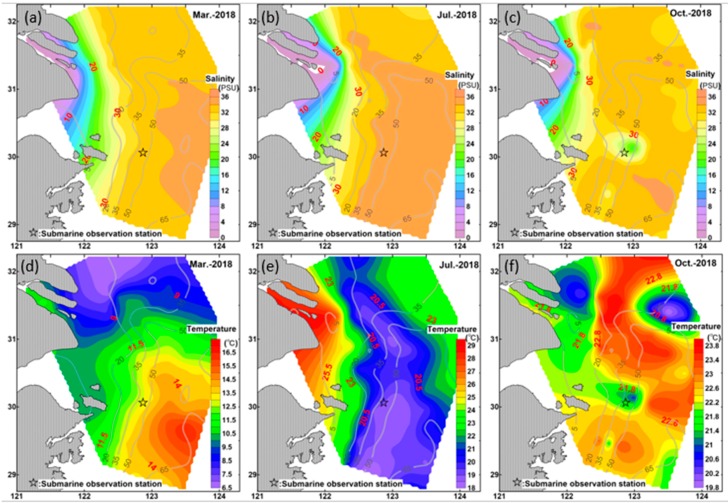
Salinity maps obtained in March, July, and October 2018 for the study region (**a**–**c**: salinity; **d**–**f**: temperature). Grey lines with grey numbers are the isobaths; red numbers are the values of the presented variable; black star indicates the location of the submarine observation.

Terrestrial input by coastal currents was the first reason we considered for its significant seasonal differences. However, the results reported by Xie et al. [45] indicated that suspended sediments in the outer areas of the Changjiang Estuary are little influenced by sediments carried by Changjiang diluted water. The complex interactions of water masses were considered the major factor affecting the conspicuous seasonal variations in suspended sediments in the ECS. As shown in Figure 1, coastal currents, including the Yellow Sea Coastal Current, the Changjiang Diluted Water and the Zhejiang and Fujian Coastal Current, and the Taiwan Warm Current, are the main currents considered to be affecting sediment variation. The field temperature and salinity data collected during the three expeditions were used to distinguish the seasonal dynamics of these currents (Figure 9).

In the flood season (May–September), due to the sharp increase in runoff from the Yangtze River into the sea, a longer high temperature and low salinity tongue occurred in the estuary (Figure 9b,e). The low-temperature and high-salinity water mass from the deep layers of the ocean in the southeast corner moved northward and merged with the Yellow Sea Cold Water Mass (YSCWM) near the mouth of Yangtze River Estuary, creating a north–south barrier preventing the horizontal transport of particulate matter. The intensified stratification weakens water vertical mixing and accelerates deposition, so sediments from continental runoff input are always trapped in nearshore areas and only few types of sediment can be transported to outer shelf [24].

In dry season, especially in winter (Figure 9a,d), the Yellow Sea Coastal Current and the Zhejiang and Fujian Coastal Current (ZFCC) were linked together by the Changjiang Diluted Water (CDW) to create a southward coastal current system, intensifying the vertical mixing of water in the coastal areas. Affected by the seabed topography and Ekman transport in the Northern Hemisphere, the low salinity surface water converges in coastal areas in winter and causes offshore transport in the bottom layer. The Taiwan Warm Current (TWC), which is characterized by high salinity and high temperature, is a weak narrow flow and its northward climb along the continental shelf is hindered in winter [46]. In other words, sediments carried by coastal waters in winter can be transported to the station, and even further areas, in accordance with proposals in earlier studies [44,47,48]. The higher concentration of suspended sediments found in February also confirms the conclusions proposed by Yang et al. [49] that sediments are mostly stored in the estuary in spring and summer and then are transported to open seas in autumn and winter.

If the assumption that variations of suspended sediments in spring tides are mostly contributed by resuspension is true, the size distribution of suspended particles seemed to be coarser than surface sediments coving the seabed. Restricted by the available data, no definitive explanation can be proposed here. The continuous records only detailed the bottom layer and covered a small area over the middle continental shelf. To study and quantify the transport and dynamics of suspended sediments in the region, more data covering a wider area, from the mouth of the estuary to the deep sea, are necessary. Vertical profiles should also be included. Instruments with a wider size range are required in future schemes for the open-ended particle size spectra presented in February.

## 5. Conclusions

Continuous data collected at the seafloor observatory provide a basis for the study of the momentum conversion and energy balance in the sea bottom boundary layer, which plays an important role in circulation structure, resuspension, and deposition of suspended matter, as well as erosion and accumulation of riverbeds. The three reclassified types of particles indicated how the particle sizes vary over time. Tidal current is the dominant factor affecting daily variations in sediments in the sea bottom layer. The maximum volume concentrations of particles were usually measured before the current velocity peaked. Considering the two seasons together, we found the widespread presence of macroflocs, which was not reported in any previous study, to the best of our knowledge. Particle concentration was found to be higher in the flood season (September) than in the dry season (February). Floc sizes are also coarser in flood seasons, from which we inferred that surface sediment at the station is coarser as well. Since all the processes, such as resuspension, advection, settling, aggregation, and disaggregation, occur together with tidal currents, we could not quantify their accurate influences separately without obtaining more data. The power law model was verified by the in situ data and was proven to be appropriate for estimating the number of particles in each particle size, accompanied by the same optimal reference *D*_0_ located at 63.11 μm. The model worked well in long-term observation and the slope of the approximation displayed obvious nearshore water characteristics, fluctuating in the range of three to four at the site.

Currents in the area are probably the major reason for the seasonal variations of sediments in the sea bottom layer. Enhanced vertical stratification and coastal upwelling along the inner shelf prevent suspended sediments from being transported through the salinity front during the flood season. However, sediments can be carried to the outer sea by downwellings and strong monsoons, contributing to the coarser particle size in February. The intensity of Taiwan Warm Current is also a crucial factor affecting the diffusion distance of sediments in the bottom layer. Wind speed has no obvious effect on suspended sediments except typhoons, when the mixing induced by cyclones is so strong that the water stratification can be broken and sediment movement can be initiated [3,50].

## Figures and Tables

**Table 1:** Several parameters derived from the harmonic analysis of tidal current in different layers (PE, percent energy of every tidal component; PTV, percent tidal variance captured by all the components included in the model).

Date	Layer ID	Component	PE (%)	Major Axis Length (cm)	Minor Axis Length (cm)	Orientation Angle (°)	Phase Lag	PTV (%)
15 September 2015 – 20 September 2015	1	K2	43.36	5.62	0.158	87.6	108	94.4
		S2	32.34	4.85	−0.0176	87.8	294	
		K1	11.48	2.89	−0.0083	126	93.2	
		P1	10.64	2.78	0.0248	125	100	
	10	K2	37.08	6.01	2.62	109	111	95.8
		S2	27.01	5.21	2.04	109	297	
		K1	16.76	4.20	−1.32	95.5	58.6	
		P1	16.24	4.15	−1.27	94.5	65.6	
4 February 2018 – 9 February 2018	1	K2	29.74	4.42	0.0349	105	48.4	95.9
		S2	27.17	4.23	−0.0892	105	326	
		P1	20.59	3.44	−1.31	109	222	
		K1	19.13	3.33	−1.22	110	131	
	10	K2	30	6.13	−2.88	18.7	43.4	96.9
		S2	24.98	5.60	−2.62	19.8	323	
		P1	21.59	5.51	−1.63	53.6	231	
		K1	19.71	5.27	−1.53	55.1	138	

**Table 2:** Variations (mean ± SD) of the selected parameters measured during the two periods. SPM1, SPM2, and SPM3 refer to single grain, microflocs, and macroflocs, respectively.

Date	VC (μL/l)	D25 (μm)	D50 (μm)	D75 (μm)	SPM1 (%)	SPM2 (%)	SPM3 (%)
15–20 September 2015	249.4 ± 53.8	91.6 ± 13.0	193.4 ± 26.3	293.5 ± 21.2	11.5 ± 1.5	24.0 ± 3.4	64.5 ± 5.5
	(142.3–536.5)	(61–147.2)	(137.1–277.1)	(232.3–356.4)	(7.1–16.1)	(15.0–34.1)	(51.5–77.1)
4–9 February 2018	158.0 ± 77.7	42.0 ± 13.0	129.9 ± 51.5	282.0 ± 79.8	24.0 ± 7.4	28.6 ± 5.8	47.3 ± 12.0
	(88.2–542.1)	(14.9–78.8)	(35.7–235.0)	(85.3–424.4)	(13.6–49.7)	(19.2–46.3)	(15.4–66.5)

**Table 3:** Results of harmonic analysis derived in sea levels and volume concentration of suspended particles. (PTV lower than 50% is shown in boldface).

Date	Type	Component	PE (%)	Amplitude (A)	Phase Lag (g)	PTV (%)
15 September 2015 – 20 September 2015	Sea level	K1	48.92	3.11	202	99.9
		P1	41.21	2.86	208	
		M2	5.53	1.05	256	
		K2	1.49	0.543	343	
	All particles	K1	31.35	1488	290.7	64.0
		P1	31.16	1483	296.0	
		K2	19.35	1169	16.11	
		S2	12.98	957	203.3	
	Single grain	K2	37.64	207	5.03	**38.8**
		S2	26.47	174	191	
		P1	15.82	134	302	
		K1	15.52	133	296	
	Microflocs	K2	49.15	462	355	**48.6**
		S2	35.44	393	182	
		P1	5.66	157	259	
		K1	4.95	147	252	
	Macroflocs	K1	40.98	1244	294	70.6
		P1	39.91	1228	300	
		K2	8.74	574	37.4	
		S2	5.57	459	226	
4 February 2018 – 9 February 2018	Sea level	K2	37.21	6.12	263	99.2
		S2	26.95	5.21	186	
		P1	15.57	3.96	44.3	
		K1	13.94	3.75	307	
	All particles	K2	46.29	2403	95.6	79.4
		S2	36.38	2131	14.8	
		P1	7.83	988	207	
		K1	7.74	983	114	
	Single grains	K2	31.15	978	55.6	75.6
		S2	24.43	866	333	
		P1	21.88	820	202	
		K1	21.14	805	110	
	Microflocs	K2	49.21	1190	103	79.1
		S2	39.16	1060	21.9	
		P1	4.81	372	214	
		K1	4.78	371	121	
	Macroflocs	K2	48.78	677	141	67.2
		S2	40.18	614	60.9	
		P1	4.23	199	21.1	
		K1	3.84	190	289	
